# *In vivo* cellular imaging of various stress/response pathways using AAV following axonal injury in mice

**DOI:** 10.1038/srep18141

**Published:** 2015-12-16

**Authors:** Kosuke Fujita, Koji M Nishiguchi, Yu Yokoyama, Yusuke Tomiyama, Satoru Tsuda, Masayuki Yasuda, Shigeto Maekawa, Toru Nakazawa

**Affiliations:** 1 Department of Retinal Disease Control, Graduate School of Medicine, Tohoku University, Sendai, 980-8574, Japan; 2 Department of Advanced Ophthalmic Medicine, Graduate School of Medicine, Tohoku University, Sendai, 980-8574, Japan; 3 Department of Ophthalmology, Graduate School of Medicine, Tohoku University, Sendai, 980-8574, Japan

## Abstract

Glaucoma, a leading cause of blindness worldwide, is instigated by various factors, including axonal injury, which eventually leads to a progressive loss of retinal ganglion cells (RGCs). To study various pathways reportedly involved in the pathogenesis of RGC death caused by axonal injury, seven pathways were investigated. Pathway-specific fluorescent protein-coded reporters were each packaged into an adeno-associated virus (AAV). After producing axonal injury in the eye, injected with AAV to induce RGC death, the temporal activity of each stress-related pathway was monitored *in vivo* through the detection of fluorescent RGCs using confocal ophthalmoscopy. We identified the activation of ATF6 and MCP-1 pathways involved in endoplasmic reticulum stress and macrophage recruitment, respectively, as early markers of RGC stress that precede neuronal death. Conversely, inflammatory responses probed by NF-κB and cell-death-related pathway p53 were most prominent in the later phases, when RGC death was already ongoing. AAV-mediated delivery of stress/response reporters followed by *in vivo* cellular imaging is a powerful strategy to characterize the temporal aspects of complex molecular pathways involved in retinal diseases. The identification of promoter elements that are activated before the death of RGCs enables the development of pre-emptive gene therapy, exclusively targeting the early phases of diseased cells.

Glaucoma, a leading cause of blindness worldwide, is characterized by a chronic and progressive loss of retinal ganglion cells (RGCs) that results in characteristic visual field defects. Although elevated intraocular pressure (IOP) plays a central role in the pathology of some forms of glaucoma, numerous clinical^1^, epidemiological^2^ and genetic studies^3^,^4^ have uncovered various IOP-independent factors associated with the disease. It is now widely accepted that most cases of glaucoma are multifactorial in origin. Among them, optic nerve compression occurring at the level of the lamina cribrosa, causing axonal injury, has been proposed as an important mechanism that triggers RGC loss^5^.

Studies using animal models have provided insights into the molecular pathologies of RGC death. Hypothesis-driven targeted investigation has revealed various genes and pathways including calpain^6^, glutamate excitotoxicity^7^ and nitric oxide synthase 2^8^ that might contribute to the pathogenesis of RGC death. At the same time, comprehensive gene expression analyses conducted to elucidate the key framework of molecular events, which defines RGC death following murine axonal injury, have been particularly informative. We recently performed comprehensive gene expression analyses of the murine retina following optic nerve crush (ONC) injury by applying two independent protocols: RNA sequencing and cap analysis of gene expression^9^,^10^. The studies identified a list of differentially expressed genes and molecular pathways that might orchestrate the pathological processes related to RGC death, including members of the ER stress pathway, p53 pathways and oxidative stress pathway. However, many of these potentially important pathways and genes require further molecular characterization to enable precise ascertainment of their roles. In particular, expression profiles of most of these “candidates” have not been studied adequately with regard to their specificity to RGCs and changes in expression levels over the course of the disease process.

AAV has been applied to numbers of studies on eyes due to its ability to stably and effectively deliver a gene of interest to the target retinal neurons^11^,^12^,^13^,^14^. Because it elicits minimal immune responses^15^ and can mediate long-term gene expression in various cell retinal types, it is now used widely in various gene therapy clinical trials for treating intractable retinal disorders^16^. With the emergence of next generation AAVs, several AAV serotypes with different capsid compositions rendering differences in targetable cell types have been used in research^11^,^12^,^13^,^14^. Particularly, AAV2/2 has been reported to transfect the RGCs through intravitreal injection effectively without infecting neurons in the middle/outer layers of the retina^14^,^17^. This feature has been exploited in studies examining the pathologies of RGCs and developing gene therapies targeting diseases affecting RGCs. In particular, glaucoma, a major cause of blindness worldwide, appears to be a good target because of its chronic progressive nature and the lack of sufficient treatment^12^,^18^,^19^. Among the different approaches proposed, one of the most promising gene therapy strategies is the delivery of a viral vector encoding neurotrophic factor including CNTF^12^.

In this study, we developed a group of pathway-specific reporters useful for quantitating activities of molecular pathways involved in retinal diseases. By combining these tools with features of optically transparent eyes that enable *in vivo* imaging of RGCs at a single-cell resolution^20^,^21^,^22^, we set out to characterize the temporal profile of selected stress/response pathways of interest in the RGCs in a mouse model of axonal injury.

## Results

### Construction and *in vitro* assessment of seven pathway reporters

**Figure 1:**
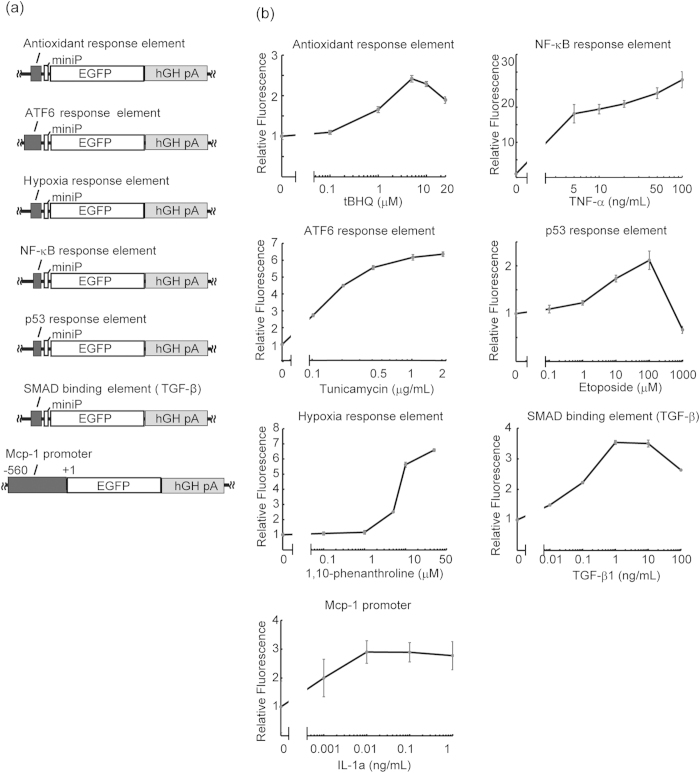
Construction and *in vitro* assessment of 7 pathway reporters (a) Schematic representation of the reporter vectors. Each stress/response promoter element was designed to drive *EGFP* reporter gene expression. MiniP, minimal promoter, which contains the TATA-box, is required for stable transcription. The six response elements do not contain the TATA-box within the sequence; thus, miniP was fused to each of the elements. Meanwhile, the Mcp-1 promoter did not require miniP as it has its own internal TATA box; hGh pA, human growth hormone polyA signal. (**b**) Results of the *in vitro* reporter assay using specific inducers. Fluorescence relative to baseline values measured in cells with no pathway inducer added is presented. Positive dose-response relations were observed for all constructs. The bars represent means ± S.E.M. (*N* = 6 each).

All the pathways evaluated were selected on the basis of two factors. First, the involvement in RGC death or neural death had been supported by solid evidence. Second, the promoter (or its element) that tags the pathway in question has been well characterized, and more importantly, is available for use. These criteria led to the selection of seven promoter elements for deriving the expression of a reporter gene, *enhanced green fluorescent protein* (*EGFP*), and to visualize various stress/response pathways. These selected elements probed oxidative stress^23^ (antioxidant response element), endoplasmic reticulum (ER) stress^24^ (ATF6 response element), hypoxia^25^ (hypoxia response element), NF-κB-mediated response^26^ (NF-κB response element), p53-mediated response^27^ (p53 response element), TGF-β-mediated response^28^ (SMAD binding element) and Mcp-1-mediated response^29^ (Mcp-1 promoter; Fig. 1a), all of which had been previously characterized by others. The constructs were then packaged into AAV2/2 for additional expression studies.

To ascertain whether each constructed reporter vector reflects the activity of a specific stress/response pathway of intention, we conducted *in vitro* experiments using reporter plasmids, HEK293T cells and known inducers of each target pathway. A detectable dose-dependent increases in EGFP fluorescence was observed for all tested reporters (Fig. 1b). Following pathway inducer administration, the maximal expression of *EGFP* gene relative to baseline measurements of the antioxidant response element, ATF6 response element, hypoxia response element, Mcp-1 promoter, NF-κB response element, p53 response element and SMAD binding was increased by a 2.28 ± 0.06, 6.36 ± 0.13, 7.14 ± 0.21, 2.89 ± 0.33, 27.80 ± 0.84, 2.12 ± 0.19 and 3.54 ± 0.22-fold, respectively. These results were consistent with reporter constructs delivered by AAV2/2 responding properly to the intended stress.

### *In vivo* imaging of stress responses in RGCs at single-cell resolution following ONC in mice

**Figure 2:**
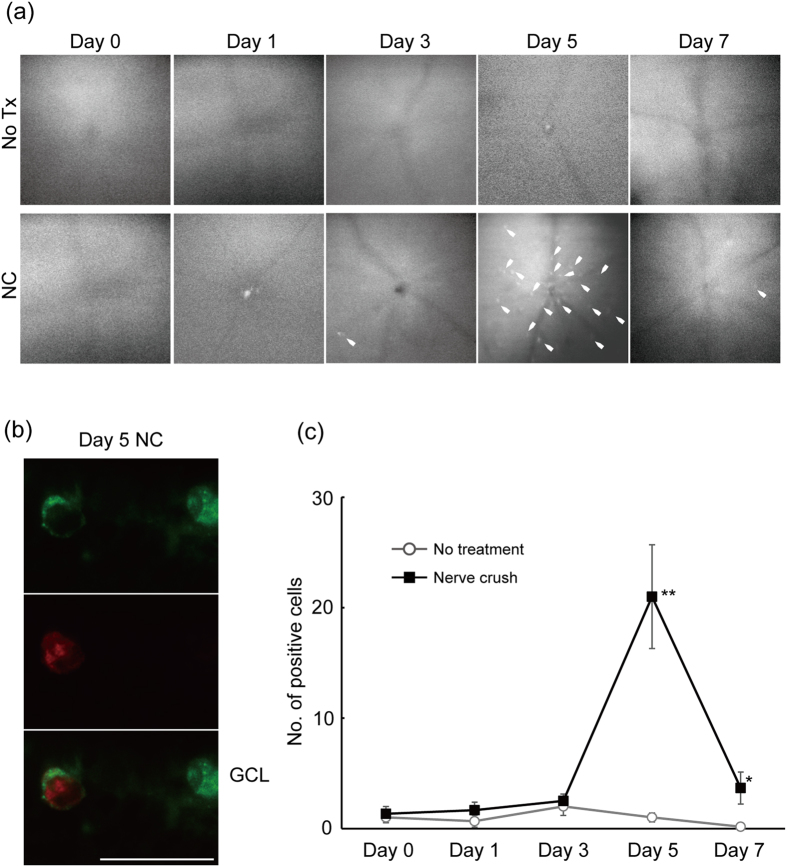
*In vivo* imaging of RGC apoptosis in murine ONC. (**a**) Representative images of apoptotic RGCs stained with Annexin V-FITC before and 1, 3, 5 and 7 days after ONC obtained using *in vivo* confocal microscopy. The upper panel shows images from the control eye, whereas the lower panel shows image from the ONC eyes. Note that Annexin V labelling and imaging were conducted on a different set of mice for each time point (*N* = 6 each) (**b**) Retinal section showing co-localization of Annexin V and cleaved caspase 3 in the RGC layer following ONC. Annexin V (green); cleaved caspase 3 (red). (**c**) Quantification of fluorescence-positive RGCs from images acquired through *in vivo* imaging of dying cells. The peak in RGC death occurred at day 5. Images were only a single picture with the optic nerve in the centre. Data represent means ± S.E.M. **P < 0.05, *P < 0.1. Scale bar: 50 μm. ONC, optic nerve crush injury; No Tx, no treatment.

As a model of RGC death, we chose the murine ONC model, which captures features of axonal injury-driven RGC death that might play a role in the pathogenesis of glaucoma^5^. First, we characterized the model by ascertaining the time course of RGC death following ONC. Dying cells were stained with Annexin V through intravitreal injection of the agent^20^,^22^. The cells were then imaged *in vivo* using confocal ophthalmoscopy, which allows the visualization of the fluorescent signal from the RGCs in the posterior pole at a single-cell resolution^20^,^21^,^22^ (Fig. 2a). A single image centred at the optic nerve was analysed for each eye. Quantification of dying RGCs at the retinal surface at 1, 3, 5 and 7 days after injury revealed an increase in cell death compared with untreated contralateral eyes by day 5 (21.00 ± 4.70 vs 1.00 ± 0.41 positive cells/view; *P* = 0.0031; Fig. 2c). The death event subsided quickly by day 7 such that increased fluorescence-positive cells were reduced (3.67 ± 1.45 vs 0.17 ± 0.15 cells/view; *P* = 0.053). The results indicate that RGC cell death peaks at day 5 after optic nerve crush, which is consistent with our earlier finding that the number of remaining RGCs decreases rapidly between day 5 and day 7 following ONC^6^.

Next, we studied the extent of AAV2/2 transduction through the intravitreal injection of AAV2/2 carrying reporter *EGFP* driven by the CMV promoter in normal mice. As expected, results showed that nearly all the *EGFP*-transduced cells at the retinal surface also expressed the RGC marker RBPMS^30^ (Supplementary Fig. 1), suggesting that the signals imaged using *in vivo* confocal imaging technique each reflect single cells, mostly RGCs. The observation was further confirmed by retrograde labelling of RGCs through an injection of Fluoro-Gold from the superior colliculus, showing that indeed EGFP-positive cells project axons to the brain, a feature associated uniquely with RGCs.

**Figure 3:**
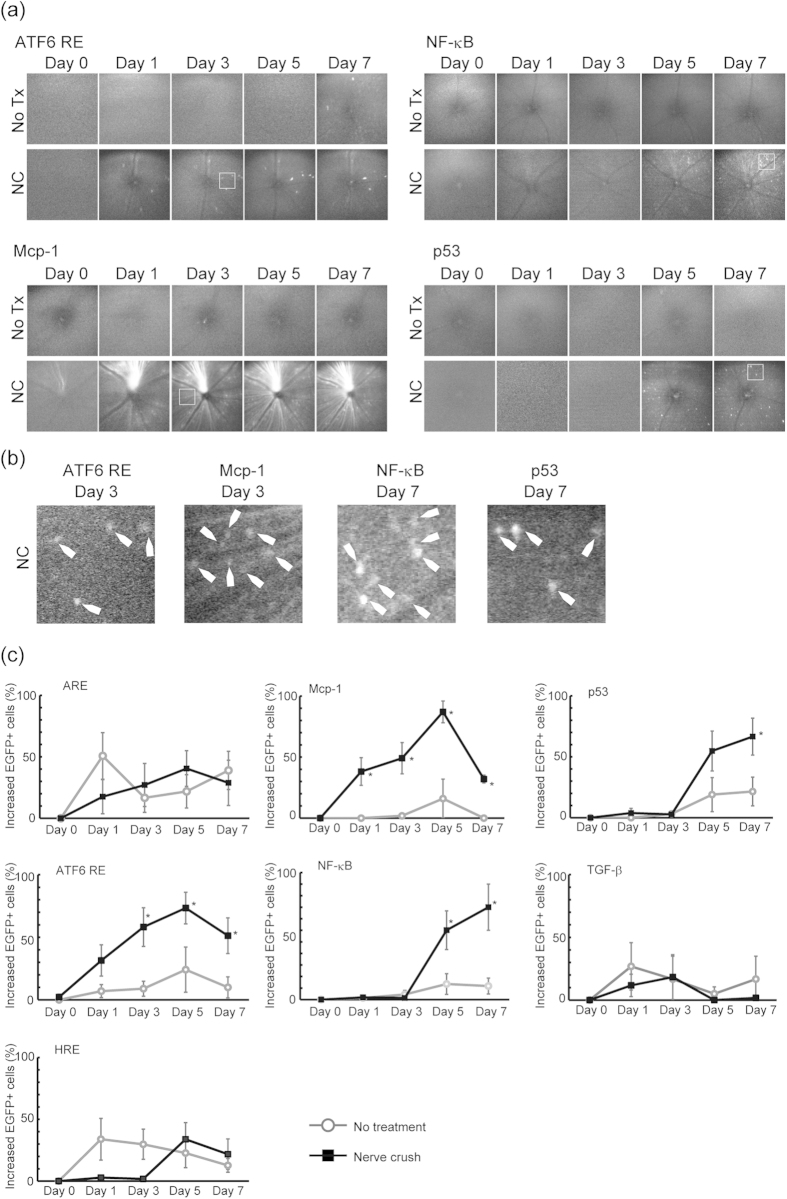
*In vivo* imaging of stress/response pathways in RGCs using AAV reporters. (**a**) Representative images of stress responses in RGCs before and 1, 3, 5 and 7 days after ONC obtained using *in vivo* confocal microscopy. Upper panels display the images from the control eye whereas the lower panels display images from the ONC eyes. (**b**) Magnified image of the inset indicated in (a). (**c**) Quantification of EGFP-positive RGCs from images acquired through *in vivo* imaging (Mcp-1 and NF-κB, *N* = 5 each; TGF-β, *N* = 6; ARE, ATF6 and p53, *N* = 7 each; HRE, *N* = 8). Data were acquired from AAV2/2 reporter-injected mice with and without ONC. For each eye, a single picture with the optic nerved in the centre was analyzed. Increased EGFP+ cells (%) was determined by dividing the increase in the number of EGFP-positive cells from baseline by the number of cells observed at the baseline. Data represent means ± S.E.M. *P < 0.05. ONC, optic nerve crush injury; No Tx, no treatment. ARE, antioxidant response element; ATF6, ATF6 response element; HRE, hypoxia response element; Mcp-1, Mcp-1 promoter; NF-κB, NF-κB response element; p53, p53 response element; TGF-β, SMAD-binding element.

Various studies have identified roles for different pathways in the pathogenesis of the RGC death^23^,^26^,^27^. Therefore, it is feasible to postulate that some of these pathways work in concert to contribute to a specific pathology, whereas others participate in different aspects of the disease; however, this point has not been addressed adequately. From this perspective, an effort to elucidate the precise activity status of a given pathway measured repeatedly in the same eyes at a single-cell resolution in relation to those of other pathways characterized in parallel is of substantial value. With this point in mind, we performed ONC in only one eye of mice that had been stably transduced with one of the AAV2/2 stress/response reporters described above, which was followed by repeated *in vivo* imaging of the EGFP-positive cells in both eyes over the 7 days following ONC. When the images centred at the optic nerve taken repeatedly at different time points were analysed, we observed three distinct patterns of pathway activation. First, increased reporter activity for ER stress and macrophage recruitment pathways, each probed by the ATF6 response element and the Mcp-1 promoter, respectively, occurred by day 3, preceding the peak in RGC death at day 5 (Fig. 3). Both pathways exhibited maximal activation at day 5, showing an increase of 73.4% ± 12.7% and 88.8% ± 11.2%, respectively, compared with baseline levels. Secondly, increased reporter activity for the NF-κB-mediated response and p53-mediated response, compared with baseline levels, was detected around day 5, and further increased at day 7 (80.0% ± 20.0% and 66.6% ± 13.2 increase, respectively; Fig. 3c). Reporter activities for the three remaining stress–response pathways, i.e. oxidative stress, hypoxia and TGF-β-mediated response, were not altered by ONC.

### Assessment of stress/response pathway by qRT-PCR and microscopic imaging

**Figure 4:**
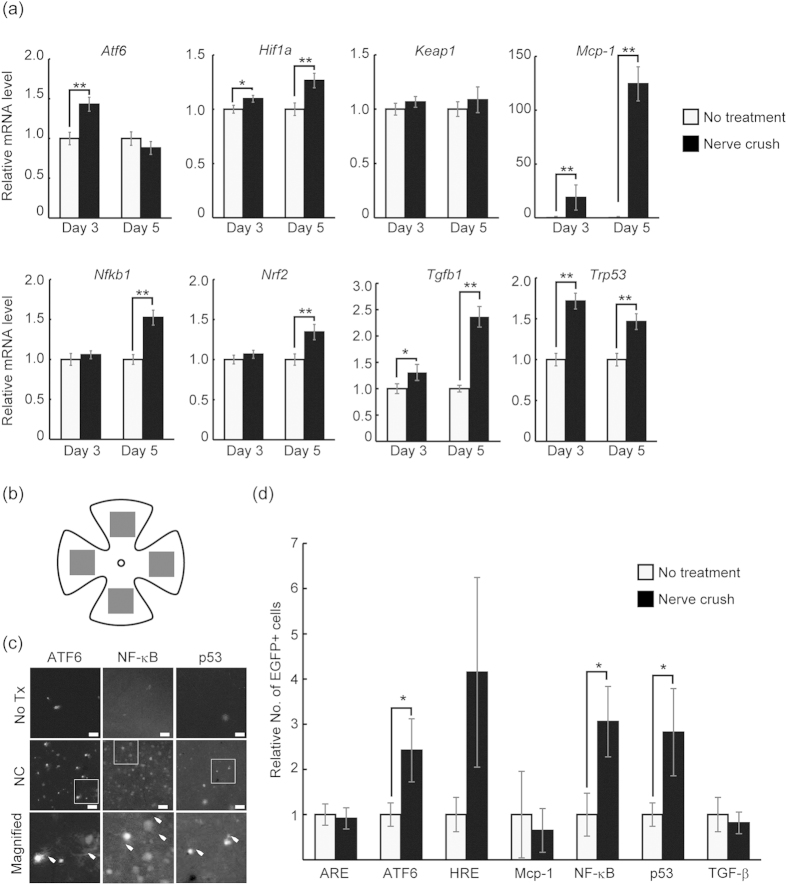
Assessment of stress/response pathways by qRT-PCR and microscopic imaging. (**a**) qRT-PCR analysis of stress-response pathway-related genes. The graphs show levels of mRNA expression in ONC eyes and the non-treated contralateral eyes. The average for the non-treated eyes was set at 1.0. The relative expression is presented for the ONC eyes (*N* = 6 each). (**b**) Schematic representation of the retinal areas imaged to quantify EGFP expression in the retinal flatmounts. EGFP-positive cells were counted in four distinct areas of 1.29 mm^2^ each (one area per retinal quadrant; filled square). (**c**) Representative images of EGFP-positive cells in the retinal flatmount imaged by conventional microscopy. EGFP-positive cells in the magnified images of the insets are labelled with arrowheads. (**d**) Quantification of EGFP-positive cells from photographs obtained by microscopic imaging of the retinal flatmounts. The average for the non-treated eyes was set at 1.0, and the relative number of EGFP-positive cells is presented for the ONC eyes (*N* = 6 each). Data represent means ± S.E.M., **P < 0.05, *P < 0.1. Scale bar: 100 μm. ONC, optic nerve crush injury; No Tx, no treatment. ARE, antioxidant response element; ATF6, ATF6 response element; HRE, hypoxia response element; Mcp-1, Mcp-1 promoter; NF-κB, NF-κB response element; p53, p53 response element; TGF-β, SMAD-binding element.

To analyse the results of the observed *in vivo* pathway response, the expression of related members of the pathway were assessed using qRT-PCR on days 3 and 5. Results showed that the mRNA levels of various stress-pathway-related genes were up-regulated at these time points in the ONC eyes compared with the untreated eyes (Fig. 4a). *Hif1a*, *Mcp-1*, *Tgf-b* and *Trp53* were upregulated at both day 3 and day 5. In addition, the Atf6 mRNA level was found to be elevated only at day 3 in contrast to *Nfkb1* and *Nrf2* mRNA, which showed an increased expression only at day 5. Furthermore, the eyes were collected at day 7. They were then processed with a view to producing retinal flatmounts that were subjected to direct microscopic evaluation (Fig. 4b–d). Quantification of the images revealed a significantly increased number of EGFP-positive cells in the ONC eyes expressing reporters driven by response elements for ATF6 (2.42 ± 0.70-fold), Mcp-1 (18.60 ± 9.00-fold), NF-κB (3.01 ± 0.78-fold) and p53 (2.82 ± 0.97-fold) compared with the control contralateral eyes.

## Discussion

This report describes the development of a simple, sensitive and versatile approach to visualize ongoing molecular events in retinal neurons at a single-cell resolution following axonal damage in mice, using AAV2/2 vectors carrying pathway-specific reporters. The integrity of the imaging data was further verified using qPCR and direct microscopic imaging of the reporters, which reinforced the method’s reliability. Because AAV2/2 expression in the retina is known to be stable over many years in mammals^31^, the technique supports repeated assessment of the molecular events in the same eye over an extended period. This unique feature is critically important when testing the effect of a new drug because early and long-term efficacy can be assessed along with the toxicity of an agent in the same group of mice, which could substantially increase the sensitivity of the experiment by overcoming the difference in drug responses between individual animals. Furthermore, by virtue of the route of AAV delivery and the cellular tropism of the AAV serotype^11^,^13^,^14^ in combination with the narrow plane of focus offered by modern imaging devices, specific cell populations in the retina can be evaluated at a single-cell resolution *in vivo*. Therefore, the same approach would be applicable for studying the stress/response pathways in any other retinal disease model.

Application of this technique to a murine ONC model revealed that ER stress and macrophage recruitment pathways shared a similar pattern of temporal response, being activated early on in the course of axonal injury, preceding the peak of RGC death occurring at day 5. Quantification of the mRNA of genes related to these pathways provided additional supportive data. Moreover, the results are consistent with our data reported from recent comprehensive gene expression studies that identified the ER stress pathway as the most significantly up-regulated stress pathway in this injury model at Day 2^9^,^10^. Any elevation in Mcp-1 expression and related pathways was not detected in either of the earlier related studies, implicating the high sensitivity of the method described in the present study. Nevertheless, the results are consistent with a previous immunohistological study, which showed that RGCs express MCP-1 in the early phases of axonal injury, which likely mediates microglia recruitment and activation at the inner retinal layer^29^. Because MCP-1 is known to cause the infiltration of microglia that responds dynamically to retinal injury^32^,^33^ the results obtained from this study show a good agreement with previous reports. Our results also showed that the pathways mediated by p53 and NF-κB shared a similar pattern of activation; they were activated at the peak of the cell apoptosis probed by Annexin V, possibly with a small delay. Because the detection of the AAV2/2 reporters requires time to translate the increased transcription to the production of reporter EGFP protein, the possible delay might merely reflect technical differences. Considering that the p53 pathway is suggested to mediate cell death following ischemia^34^, excitotoxicity^27^, axotomy^35^,^36^ and ONC^37^, and that the NF-κB-driven inflammation accelerates neural apoptosis^38^, it is possible that these pathways are important drivers of RGC death. Meanwhile, RGC injury induced by a mechanism more specific to glaucoma, e.g., ocular hypertension, likely shows a different pattern of stress response. Nonetheless, several pathways, including the ER stress^39^,^40^ and p53^41^ pathways examined in this study are implicated also in this injury model. Specifically, both the ER stress pathway and the p53 pathway tagged by *Gadd45a* were found to be activated from the early injury phases, staying up-regulated for a prolonged period of time. It would be interesting to apply the methods developed in this work to understand the temporal profiles of other types of stress pathway activation in the ocular hypertension model and in other models of RGC damage.

It appears plausible that some differences exist between the results of *in vivo* reporter imaging and those of qPCR. For example, *in vivo* imaging showed no alteration of HIF1A promoter activity, whereas *Hif1a* expression was increased at day 3 and day 5 in the ONC eyes. A similar discrepancy was noted for the TGFB response element and *Tgfb1* expression. Such a discrepancy might, therefore, stem from methodological differences; qPCR evaluates mRNA expression in the whole retina sample as opposed to *in vivo* imaging, which targets RGCs. The results of reporter assays also depend on the transcription efficacy of the sub-cloned promoter elements, which are usually less efficient than the longer native genomic promoter. Moreover, the reporter assay and qPCR analysis of a given pathway do not necessarily reflect the activity of the same gene. For example, the hypoxia pathway evaluated by the reporter assay probes the transcription efficacy of the hypoxia response element, which might reflect the activity of both HIF1A and HIF2A^42^ and perhaps other genes, whereas qPCR analysis of hypoxia evaluates the expression of a single representative gene. Meanwhile, a quantitative comparison of EGFP-positive cells between the ONC eyes and the control eyes at day 7 showed similar results for both *in vivo* imaging and microscopic analysis of the retinal flatmounts.

In previous studies, the temporal profile of RGC death following ONC indicated that RGC death continues after 7 days^43^,^44^. Initially, these results may appear somewhat different from the cell death profile detected using Annexin V in this study. However, direct comparison of the data is difficult due to a few important differences in the methodology. First, unlike the conventional quantification of RGCs, which is typically achieved through counting the surviving cells, the current method probes a small number of cells in a particular phase of death. Second, the current method evaluates only the posterior pole of the retina, the area around the optic nerve, whereas the area outside the posterior pole was also evaluated in previous histology-based methods. Third, the number of cells that are quantifiable may be slightly altered by different imaging approaches. For example, retrograde labelling of RGC from the superior colliculus may not label a set of RGC that projects only through LGN. Similarly, immunohistochemistry may be influenced by the specificity of the antibody to particular groups of RGCs that are known to comprise at least 25 different cell types^45^ on the basis of morphology and receptive field properties. Finally, at least in our hands, the decrease in residual RGC number was most prominent at around days 5 and 7 after ONC, which was followed by a much more gradual loss of RGC until day 14 as evidenced by retrograde labelling of RGC from the superior colliculus^6^. This is not incompatible with increased RGC death at day 5 followed by a decline at day 7 as detected by *in vivo* imaging in this study.

The results of this study also offer important potential therapeutic perspectives. The pathways shown to be up-regulated can themselves be suitable targets for neuroprotection of the RGCs. Translational utilities also include the application of promoter elements to gene therapy. For example, two promoter elements, the ATF6 response element and the Mcp-1 promoter, were actively transcribed before the peak in RGC death in the ONC eyes. These promoter elements can be coupled with a gene encoding a reported neuroprotective factor for RGCs such as *BDNF*^46^ or *NRF*^23^,^47^ and can be packaged into AAV to develop a vector for temporally regulated gene therapy. Such treatment, which facilitates targeted expression of therapeutic molecules only in the necessary neurons and when necessary, is expected to be useful to reduce the risk of side effects that might occur because of unregulated expression of the molecule.

The limitations of the imaging platform described herein include the small area of the retina analysed, the small number of pathways analysed biased by the availability of characterized promoter elements, differences in transcription efficiency between promoters that are integral to the artefact arising from the cloned promoter or response element, and a gap in time between the event of promoter transcription and reporter expression that happens only after the translation of the mRNA and oligomerization of the amino acids.

In conclusion, we developed AAV reporters that enable the visualization of various stress/response pathways in specific retinal neurons at a single-cell resolution. The tool, which offers a convenient and powerful means to assessing the effects of therapeutic agents, can be applied to studying various pathologies in different retinal cell types and retinal diseases. The ATF6 response element and Mcp-1 promoter, both actively transcribed before the peak in RGC death, can, therefore, be used for temporally regulated gene therapy for treating RGC death caused by axonal injury.

## Methods

### Animals

This study used adult (6–12-week-old) male C57BL/6J mice (Japan SLC Inc., Hamamatsu, Japan). Surgical procedures were performed under deep anaesthesia induced by intraperitoneal administration of a mixture of ketamine (37.5 mg/kg) and medetomidine (0.625 mg/kg). The medetomidine effect was reversed by intraperitoneal injection of 1.25 mg/kg atipamezole. All mice were handled and maintained in accordance with the ARVO Statement guidelines for the Use of Animals in Ophthalmic and Vision Research, the Declaration of Helsinki, and intramural guidelines for the care and use of animals. All experimental procedures were conducted after approval by the ethics committee for animal experiments at the Tohoku University Graduate School of Medicine.

### Axonal injury

As described previously^9^, ONC was performed to induce axonal injury in RGCs. In brief, the optic nerve was exposed, avoiding the blood vessel, and crushed approximately 2 mm posterior to the eyeball with forceps for 5 s. Several minutes after ONC, the recovery of retinal circulation was confirmed by fundus examination. Antibiotic ointment was applied to the treated eye.

### Immunohistochemistry

The eyes were fixed in 4% paraformaldehyde, embedded in OCT compound (Sakura Finetek Inc., Japan), and sectioned using a cryostat (model CM3050; Leica Microsystems, Germany). The section was blocked further with 5% goat serum for 30 min, incubated with rabbit anti-RBPMS antibodies (ab194213, 1: 1000; Abcam plc., Cambridge, UK) or rabbit anti-cleaved caspase 3 antibodies (#9661, 1:600; Cell Signaling Technology, Inc., Beverly, MA) for 1 h and stained with secondary antibodies (anti-rabbit Alexa Fluo 568; Invitrogen Corp., Carlsbad, CA) and 4′,6-diamidino-2-phenylindole (DAPI; Vector Laboratories Inc.) for an additional 45 min.

For retinal flatmount analysis, the eyes were fixed in 4% paraformaldehyde, and blocked with 5% donkey serum for 30 min, incubated with rabbit anti-RBPMS antibodies (ab194213, 1: 1000; Abcam plc., Cambridge, UK) for 1 h and stained with secondary antibodies (anti-rabbit Alexa Fluo 568; Invitrogen Corp., Carlsbad, CA) and 4′,6-diamidino-2-phenylindole (DAPI; Vector Laboratories Inc.) for additional 45 min. The samples were mounted onto glass slides with the RGC side facing the cover slip.

### Retrograde labelling of RGCs from the superior colliculus

The retrograde tracer Fluor-Gold 2% (FG; Fluorochrome, LLC, Denver, CO) was dissolved in saline, and labelling was performed as described previously^6^,^7^. In brief, the animals were anaesthetized and the skin over the cranium was incised to expose the scalp while the head was fixed with an ear bar. FG solution (1 μL) was injected into the superior colliculus using a 32-G needle. Seven days after labelling, the mice were killed and the eyes were fixed in 4% paraformaldehyde. Isolated retinas were fixed and flatmounted onto glass slides as described above.

### AAV reporter construction

For the reporter experiments, a 720-bp fragment of *EGFP* cDNA (Clontech, Mountain View, CA) was sub-cloned into a pAAV-MCS Promoterless Expression Vector (Cell Biolabs Inc., San Diego, CA). Promoter fragments of the antioxidant response element (Promega Corp., Madison, WI), ATF6 response element (Promega Corp., Madison, WI), hypoxia response element (Promega Corp., Madison, WI), Mcp-1 promoter (560 bp immediately upstream of the initiation codon ATG; Accession number U12470)^48^, NF-κB response element (Promega Corp., Madison, WI), p53 response element (p53 RE; Promega Corp., Madison, WI), and SMAD-binding element (Promega Corp., Madison, WI) were each sub-cloned into AAV reporter vectors without a promoter. AAV2/2 containing the reporter constructs were generated and purified following the method described previously^49^. Each virus (1 × 10^12^ gc/mL) was injected (2 μL/injection) into the vitreous of a mouse.

### *In vitro* reporter assay

HEK293T cells were maintained in DMEN + GlutaMAX-I medium (Life Technologies Inc., Carlsbad, CA) supplemented with 10% foetal bovine serum (Life Technologies Inc., Carlsbad, CA) and antibiotic–antimycotic mix (Life Technologies Inc., Carlsbad, CA), and cultured under 5% CO2 at 37 °C.

Ten microliters of DMEN + GlutaMAX-I medium containing 247.5 ng of polyethyleneimine (Polysciences, Inc., Warrington, PA), 100 ng of reporter plasmid, and 10 ng of ptd-tomato-N1 (Clontech, Mountain View, CA) was added to HEK293T cells (2 × 10^4^). Stress inducers were added to transfected cells 24 h post-transfection and were further cultured for 18 h, except for IL-1a, which was cultured for only 8 h. Three cycles of freeze–thawing in PBS were applied in order to lyse the cells. Fluorescence (excitation 485 nm/emission 535 nm and excitation 544 nm/emission 590 nm) was measured using a microplate reader (Spectra MAX GEMINI; Molecular Devices Corp., Sunnyvale, CA). Etoposide (Merck and Co. Inc., Darmstadt, Germany), hTGF-b1 (Sigma-Aldrich Corp., St. Louis, MO), IL1a (Merck, Darmstadt, Germany), 1,10-phenanthroline (Wako Pure Chemical Industries Ltd., Osaka, Japan), tert-butylhydroquinone (tBHQ; Sigma-Aldrich Corp., St. Louis, MO), tumour necrosis factor-α (TNF-α; Sigma-Aldrich Corp., St. Louis, MO) and tunicamycin (Merck and Co. Inc., Darmstadt, Germany) were used to activate p53, TGF-β, Mcp-1, hypoxia, antioxidant, NF-κB and ER stress pathways, respectively.

### *In vivo* cellular imaging

Cell death labelling was performed using Annexin V-FITC (Enzo Life Sciences Inc., Farmingdale, NY) as previously described^20^,^22^ with a slight modification. Annexin V-FITC (1 mg/mL) was injected (1 μL/injection) intravitreally. Images were acquired using confocal ophthalmoscopy 6 h post-injection. For reporter analysis, mice were injected with an AAV2/2 reporter, with subsequent ONC induction in only one eye 4 weeks later and *in vivo* imaging at days 1, 3, 5 and 7 after injury. *In vivo* cellular imaging in mice was performed using confocal ophthalmoscopy (Nidek F10; Nidek Co. Ltd., Gamagori, Japan). The animals were anaesthetized and pupils were dilated with 2.5% phenylephrine and 1.0% tropicamide. Blue laser (488 nm) was used for excitation and a band path filter set at 510–550 nm was used to capture the emission from the EGFP and Annexin V-FITC. The focus was adjusted at the RGC layer in the posterior pole immediately posterior to the surface retina. Six images were taken and averaged to obtain a single image of the posterior pole with the optic nerve head aligned in the centre. The number of fluorescent cells were quantified from a single image for a given time point per mouse.

### Quantitative RT-PCR

Extraction of total RNA was conducted using the miRNeasy mini kit (Qiagen Inc., Hilden, Germany) from the retina according to the manufacturer’s instructions. One microgram of total RNA was reverse-transcribed in a 10 μL reaction mixture using SuperScript III (Life Technologies Inc., Carlsbad, CA), and quantitative RT-PCR was performed (7500 Fast Real-Time PCR System; Life Technologies Inc., Carlsbad, CA). The 20 μL mixture, including 1 μL of cDNA, 1 μL of TaqMan probe (Life Technologies Inc., Carlsbad, CA) and 1x TaqMan Fast Universal PCR Master mix (Life Technologies Inc., Carlsbad, CA) was placed in 96-well plates. PCR was performed with an initial denaturation step of 95 °C for 20 s, followed by 40 cycles at 95 °C for 3 s and 60 °C for 20 s. For relative comparison of gene expression, we analysed the results of the qRT-PCR data using the comparative Ct method (2- ΔΔCT), normalized to *Gapdh*, an endogenous control. TaqMan probes were also used included the following ones: *Atf6* (Mm01295317; Life Technologies Inc.), *Gapdh* (Mm01256744; Life Technologies Inc.), *Hif1a* (Mm00468878; Life Technologies Inc.), *Keap1* (Mm00497268; Life Technologies Inc.), *Mcp1* (Mm00441242; Life Technologies Inc.), *Nfkb1* (Mm00476379; Life Technologies Inc.), *Nrf2* (Mm00477786; Life Technologies Inc.), *Tgfb1* (Mm00441729; Life Technologies Inc.), and *Trp53* (Mm01731290; Life Technologies Inc.).

### Retinal flatmounts and cell counting

After *in vivo* imaging (day 7), the eyes were immersed in 4% paraformaldehyde for 2 h, after which retinas were mounted onto glass slides as flatmounts. EGFP-positive cells were counted in four distinct areas of 1.29 mm^2^ each (one area per retinal quadrant; Fig. 4b) under a fluorescence microscope (Axiovert 200; Carl Zeiss Inc., Oberkochen, Germany).

### Statistical analysis

Statistically significant differences between two groups were assessed using a Student *t*-test. A probability (*P*) value of <0.05 was inferred as significant. The Grubbs–Smirnov test (*P* < 0.01) was applied to the microscopic quantification data of EGFP-positive cells to reduce the effect of false-positive signal generated during tissue handling (Fig. 4d). All values are expressed as means ± standard errors of the mean.

## Additional Information

**How to cite this article**: Fujita, K. *et al.* *In vivo* cellular imaging of various stress/response pathways using AAV following axonal injury in mice. *Sci. Rep.* **5**, 18141; doi: 10.1038/srep18141 (2015).

## Supplementary Material

Supplementary Information
