# Supplementary material for: In vivo cellular imaging of various stress/response pathways using AAV following axonal injury in mice
Source: Sci Rep. 2015 Dec 16;5:18141. doi: 10.1038/srep18141 (PMC4680972; doi:10.1038/srep18141)
Supplement: Supplementary Information [file srep18141-s1.doc]

Supplementary information for *Scientific Reports*

***In vivo* cellular imaging of various stress/response pathways using AAV following axonal injury in mice**

Kosuke Fujita^1^, Koji M Nishiguchi^2^, Yu Yokoyama^3^, Yusuke Tomiyama^3^, Satoru Tsuda^3^, Masayuki Yasuda^1^, Shigeto Maekawa^1^, Toru Nakazawa^1,2,3, *^

^1^Department of Retinal Disease Control, Graduate School of Medicine, Tohoku University, Sendai, 980-8574, Japan

^2^Department of Advanced Ophthalmic Medicine, Graduate School of Medicine, Tohoku University, Sendai, 980-8574, Japan

^3^Department of Ophthalmology, Graduate School of Medicine, Tohoku University, Sendai, 980-8574, Japan

^*^Correspondence author:

Phone: +81-22-717-7294, Fax: +81-22-717-7298

E-mail: [ntoru@oph.med.tohoku.ac.jp](mailto:ntoru@oph.med.tohoku.ac.jp)


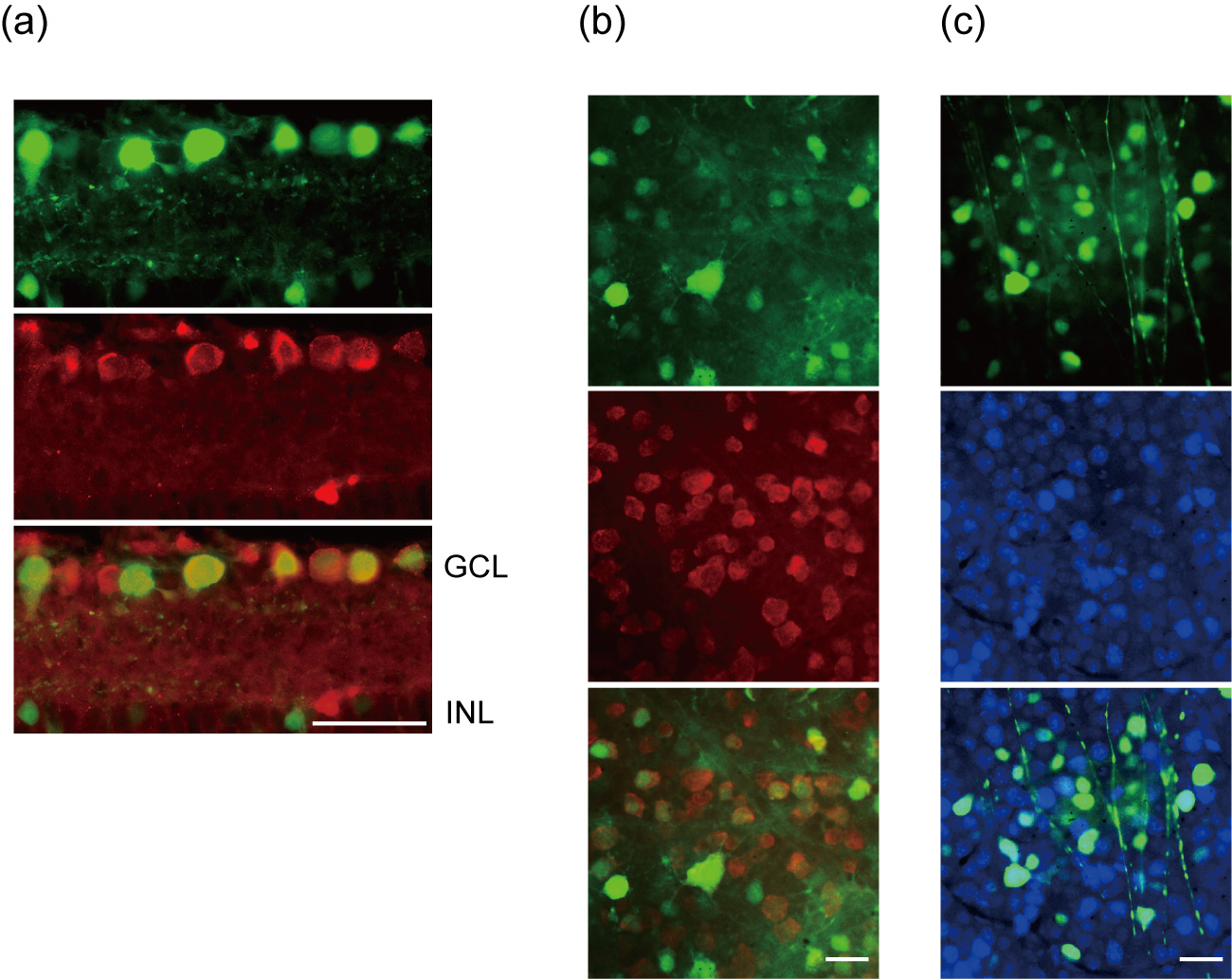


Supplementary Fig. 1

**Transduction of RGCs through intravitreal administration of AAV2/2**

1. Representative frozen section images of reporter EGFP (green) delivered by intravitreal injection of AAV2/2 (CMV promoter) stained with anti-Rbpms antibodies (red) that indicate RGCs. (b) Representative images of a retinal flatmount. Distribution of AAV2/2 reporter EGFP-positive cells (green) and Rbpms-positive RGCs (red) are shown. (c) Representative retinal flatmount images. Distribution of AAV2/2 reporter EGFP-positive cells (green) and FG-labelled RGCs (blue) are shown. In both (b) and (c), EGFP shows co-localization with RGCs. Scale bar: 50µm.
